# Comparative genomics of *Helicobacter pullorum* from different countries

**DOI:** 10.1186/s13099-020-00394-1

**Published:** 2020-12-10

**Authors:** Guilan Zhou, Hao Liang, Yixin Gu, Changyan Ju, Lihua He, Pengbo Guo, Zhujun Shao, Jianzhong Zhang, Maojun Zhang

**Affiliations:** 1 State Key Laboratory for Infectious Disease Prevention and Control, Collaborative Innovation Center for Diagnosis and Treatment of Infectious Diseases, National Institute for Communicable Disease Control and Prevention, Chinese Center for Disease Control and Prevention, Rd155, Changbailu, Changping, Beijing, 102206 People’s Republic of China; 2 Nanshan Center for Disease Control and Prevention, Shenzhen, China; 3 School of Public Health, Shandong University, Shandong, China

**Keywords:** *H. pullorum*, Comparative genomics, Genomic population structure, Virulence factors, Drug resistance genes

## Abstract

**Background:**

*Helicobacter pullorum* commonly colonized in the gastrointestinal tract of poultry and caused gastroenteritis. This bacterium could be transmitted to humans through contaminated food and caused colitis and hepatitis. Currently, the genetic characteristics of the *H. pullorum* were not recognized enough. In this study, the genomes of 23 *H. pullorum* strains from different counties were comparatively analyzed. Among them, *H. pullorum* 2013BJHL was the first isolated and reported in China.

**Results:**

The genomes of the studied strains were estimated to vary from 1.55 to 2.03 Mb, with a GC content of ~ 34%. 4064 pan genes and 1267 core genes were obtained from the core-pan genome analysis using the Roary pipeline. Core genome SNPs (cg-SNPs) were obtained using Snippy4 software. Two groups were identified with the phylogenetic analysis based on the cg-SNPs. Some adhesion-related, immune regulation, motility-related, antiphagocytosis-related, toxin-related and quorum sensing related genes were identified as virulence factors. *APH(3′)*-*IIIa, APH(2′’)*-*If,* and *AAC(6′)*-*Ie*-*APH(2′’)*-*Ia* were identified as antibiotic resistance genes among the *H. pullorum* genomes. *cat, SAT*-*4* and *tetO* genes were only identified in 2013BJHL, and *tet(C)* was identified in MIT98-5489. MIC determination revealed that the 2013BJHL showed acquired resistance to ciprofloxacin, nalidixic acid, tetracycline, gentamicin, streptomycin and erythromycin, only sensitive to ampicillin. The antibiotic resistance genetic determinants on the 2013BJHL genome correlate well with observed antimicrobial susceptibility patterns. Two types of VI secretion system (T6SS) were identified in 52.2% (12/23) the studied strains.

**Conclusion:**

In this study, we obtained the genetic characteristics of *H. pullorum* from different sources in the world. The comprehensive genetic characteristics of *H. pullorum* were first described. *H. pullorum* showed highly genetic diversity and two sub-types of T6SSs were first identified in *H. pullorum*. 2013BJHL was found to be multidrug resistant as it was resistant to at least three different antibiotic classes.

## Background

*Helicobacter pullorum* (*H. pullorum*) was a kind of Gram-negative and urease-negative bacterium, which initially isolated from the liver and intestinal of asymptomatic poultry [21]. The *H. pullorum* could be transmitted to humans through contaminated meat and it had been recognized as human pathogen which was associated with human colitis and hepatitis [5, 21, 22]. In these days, human recurrent diarrheal, Crohn’s disease and bacteremia were found to be related to the *H. pullorum* infection [3, 12, 21, 23, 24]. With the increased consumption of the chicken, *H. pullorum* might be one important emerging food-borne pathogen in the world. As the same time, isolates of poultry origin showed resistance to ciprofloxacin, gentamycin, erythromycin and tetracycline and was susceptible to colistin sulfate and ampicillin, and phenotypic resistance was almost the same as genotype resistance [4, 8, 9], however, there was no antibiotic recommendation for this organism. There were reports about the prevalence of *H. pullorum* in poultry but the positive ratios were variant (from 23.5 to 100%), which might be due to differences in detection methods and the isolation samples [4, 8, 9, 14, 25]. The isolation in poultry caecum seemed higher than in liver [1, 6]. Currently, only few of genetic characteristics of *H. pullorum* were described, and showed diversity or similarity in different sources [4, 18], but it was unclear in China. In this study, we attempted to characterize the *H. pullorum* strain isolated from retail chicken liver in China in the aspect of core-pan genome, the antibiotic resistance potential, virulence factor genes and population structure based on phylogeny. The comparative genomics of 22 *H. pullorum* strains from different sources and different countries released from NCBI to date also were investigated.

## Results

### Genomic and antimicrobial susceptibility characteristics of strain 2013BJHL

The genome size of strain 2013BJHL was 1,875,659 bp, 34 scaffolds and GC content was 34.30%. 1939 genes were predicted and the total length of genes was 1,705,674 bp, which made up 90.94% of genome. A 7.3 kb incomplete phage-associated region was identified using online software PHASTER, which consisted of genes encoding metabolism, biosynthesis and hypothetical proteins. MIC determination revealed that 2013BJHL was resistant to ciprofloxacin, nalidixic acid, tetracycline, gentamicin, streptomycin and erythromycin but only sensitive to ampicillin.

### Comparative genomic and genetic population structure

**Fig. 1:**
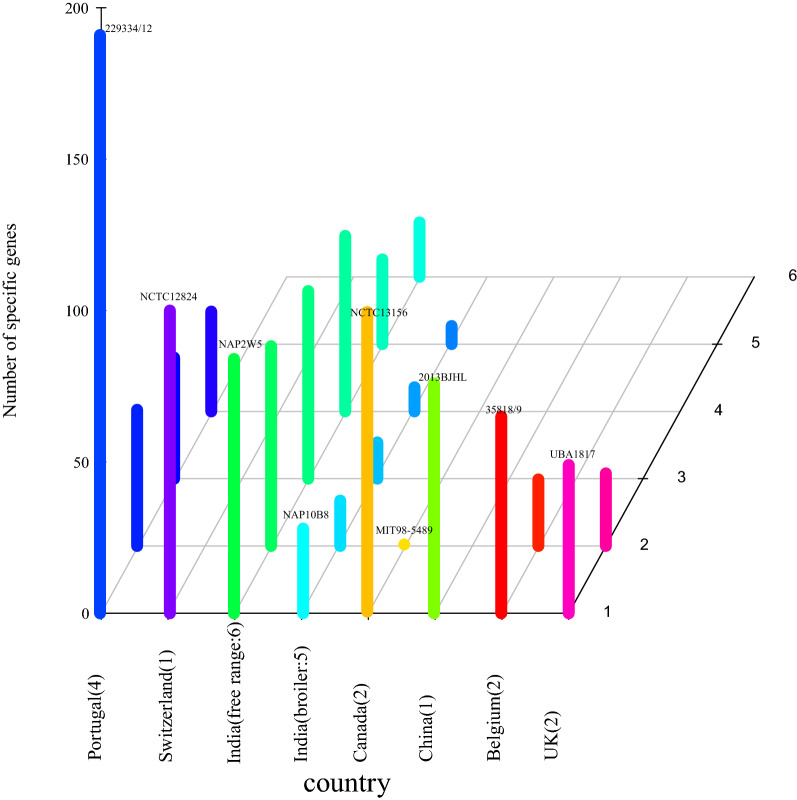
Number of specific genes in *H. pullorum.* The x-axis represents the country name and the number of strains, the y-axis represents the strain name and the z-axis represents the number of specific genes

The genomes of the studied strains were estimated to vary from 1.55 Mb to 2.03 Mb for each, with a GC content of ~ 34%. 4064 pan genes and 1267 core genes were obtained from these 23 *H. pullorum* genomes. From the pan-genome analysis, we found that the strain (ID:229334/12) from fresh chicken meat in Portugal contained the most specific genes (191 genes), followed by strain NCTC12824 isolated from chicken faeces in Switzerland (100 genes). However, human strain MIT98-5489 had the fewest specific genes (0 gene). 76 specific genes were detected in strain 2013BLHL and 71.1% (54/76) of them were predicted to be hypothetical proteins. The distribution of the specific genes in different strains was shown in Fig. 1. We also found that free-range chickens had more specific genes than broilers in Indian. The individual genes for each strain were presented in the Additional file 1.

**Fig. 2:**
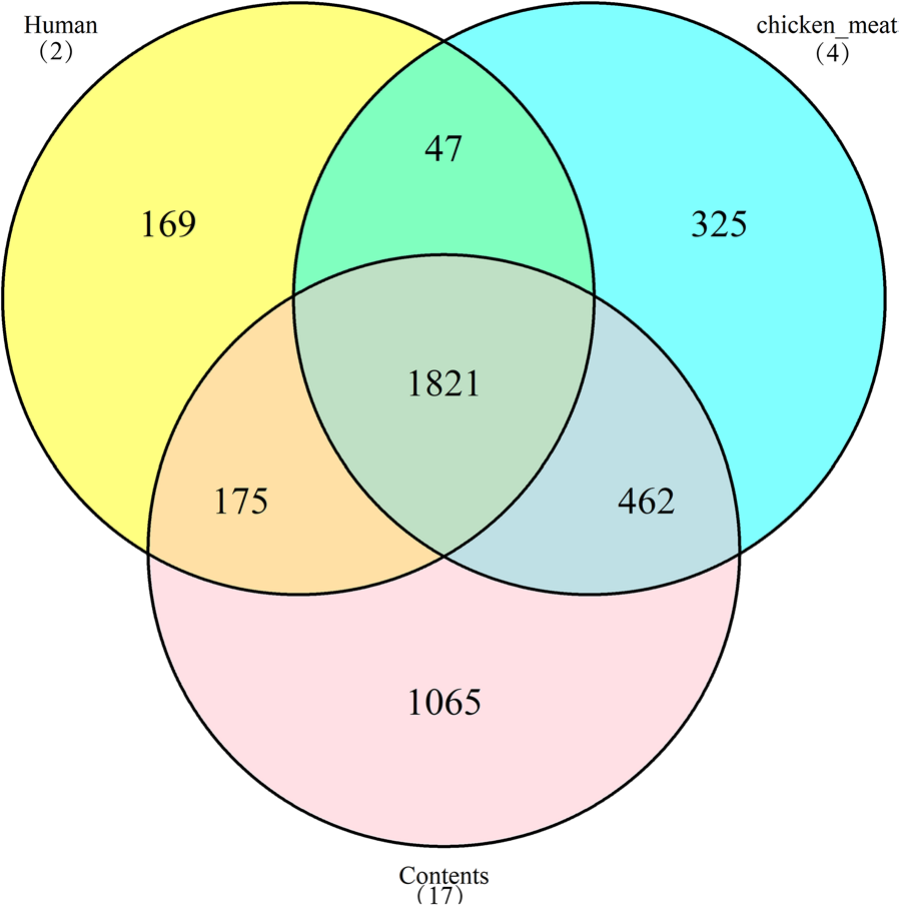
Differences in the number of genes in *H. pullorum* from different sources. The yellow circle represents the source of human (2 strains), the blue circle represents the source of chicken meat (4 strains), and the pink circle represents the source of contents (faces, caecum and liver, 17 strains). The middle common region is the core genes. The digit indicates the number of genes

According to the isolation sources, these 23 strains were classified into three groups. These three groups share 1821 genes, and the human source group had the least number of specific genes (169 genes, Fig. 2). The specific genes in each group were present in the Additional file 2. Most of the human sources known functions specific genes were transferases associated genes.

**Fig. 3:**
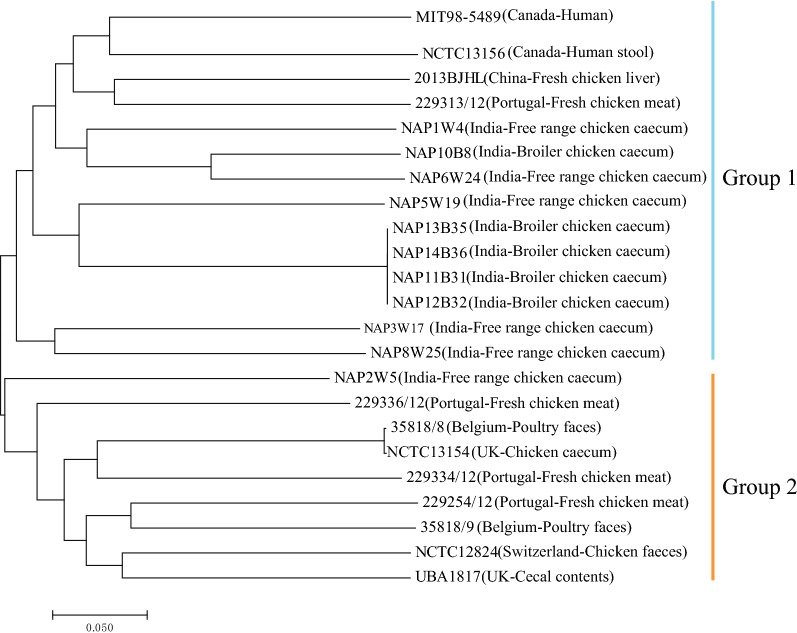
Phylogenetic tree based on core-SNPs of *H. pullorum* using MEGA7 software. The scale bar represents substitution per site

A phylogenetic tree was built based on the cg-SNPs (Fig. 3). Two groups were identified. Most of the strains in Group2 were isolated from European. However, in Group1, the strains were isolated from Indian, China, Portugal and Canada.

### Virulence, antibiotic resistance and secretion system analysis

**Fig. 4:**
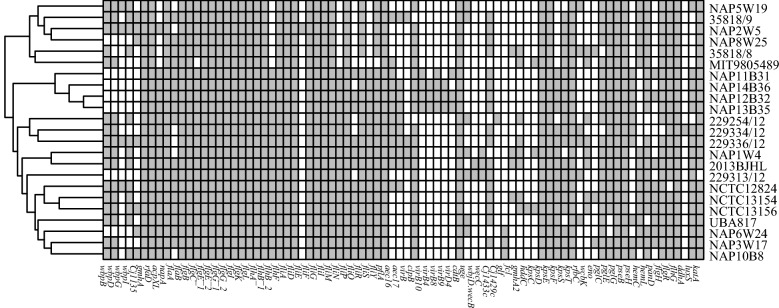
Heatmap is generated using the pheatmap package based on the distribution of virulence genes. Grey indicates the presence of the virulence genes, white indicates the absence of the virulence genes

Numerous of virulence associated genes were identified in this study: the adhesion-related genes(*wbp*), immune regulation genes(*napA*), motility-related genes(*fla*), toxin-related genes(*cdt*), antiphagocytosis-related genes(*wec*) and quorum sensing genes(*luxS*). Some of these genes were homologous with the genes in *C. jejuni,* like immune evasion, motility related genes and glycosylation system related genes. The details of the virulence genes were listed in the Additional file 3 and presented in Fig. 4.

The aminoglycoside antibiotic resistance genes AAC(6′)-Ie-APH(2′′)-Ia was found in 4 of 23 strains (17.4%, NAP11B31, NAP12B32, NAP13B35, and NAP14B36), APH(2′′)-If was found in 1 of 23 strains (4.3%, 2013BJHL) and APH(3′)-IIIa was found in 3 of 23 strains (13.0%, 2013BJHL, NAP5W19, and NAP6W24). *tet(C)* was identified in 1 of 24(4.3%, MIT98-5489). *cat, SAT*-*4* and *tetO* genes were only identified in the Chinese strain 2013BJHL, which conferred chloramphenicol, streptomycin and tetracycline resistance, respectively. With the exception of two human strains, the strains containing the drug resistance genes were isolated from Asia. *GyrA* gene individual missense mutations was detected in all strains, except 229336/12, 35818/8, 35818/9, NCTC13156, NCTC12824, NCTC13154 and UBA1817.

**Fig. 5:**
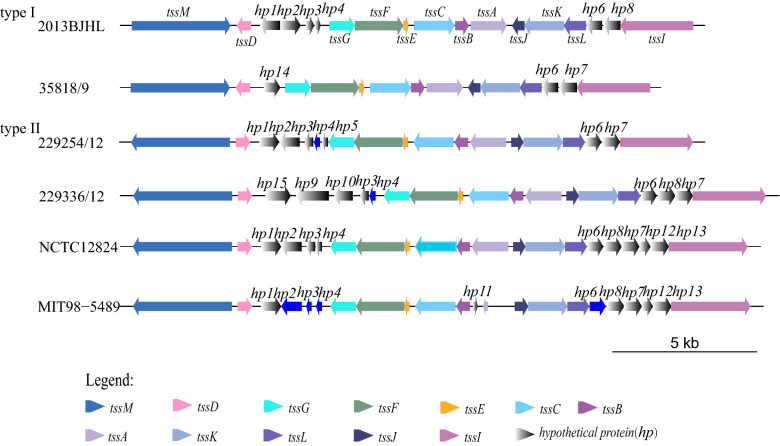
Gene arrangement of T6SS in *H. pullorum.* Different colors represent different genes, and gray represents hypothetical proteins. The length of the arrow indicates the size of the gene, and the direction indicates the direction of the gene

T6SS was identified in 52.2% (12/23) the studied strains (see Table 2 for detail). According to the identified gene arrangement, these T6SS could be classified into two types. Type I including 15–18 genes and had 18,520–20,025 bp long, Type II contained 19–22 genes and 20,056–22,572 bp long. The gene contents of each T6SS type were listed in Table 1. The gene arrangements and gene components of these two T6SS types were presented in Fig. 5.

**Table 1:** The genes contents of T6SS in *H. pullorum*

T6SS genes	Predicted functions	Identity	E-value	Accession in NCBI
tssM	Type VI secretion system membrane subunit	100.00%	0.00E+00	WP_060660915.1
*tssD*	Type VI secretion system membrane subunit	100.00%	1.00E−125	WP_005020432.1
*tssG*	Type VI secretion system membrane subunit	100.00%	0.00E+00	WP_005020437.1
*tssF*	Type VI secretion system membrane subunit	100.00%	0.00E+00	WP_060660919.1
*tssE*	Type VI secretion system membrane subunit	100.00%	6.00E−38	WP_065836879.1
*tssC*	Type VI secretion system membrane subunit	100.00%	0.00E+00	WP_005020440.1
*tssB*	Type VI secretion system membrane subunit	100.00%	0.00E+00	WP_005020441.1
*tssA*	Type VI secretion system membrane subunit	100.00%	0.00E+00	WP_060660920.1
*tssJ*	Type VI secretion system membrane subunit	100.00%	1.00E−101	WP_054197454.1
*tssK*	Type VI secretion system membrane subunit	100.00%	0.00E+00	WP_060660921.1
*tssL*	Type VI secretion system membrane subunit	100.00%	0.00E+00	WP_060660922.1
*tssI*	Type VI secretion system membrane subunit	100.00%	0.00E+00	WP_082230383.1
*hp1*	Hypothetical protein	100%	1.00E−169	WP_060660916.1
*hp2*	Hypothetical protein	100%	4.00E−177	EEquation 62550.1
*hp3*	Hypothetical protein	98.46%	2.00E−37	WP_060660917.1
*hp4*	Hypothetical protein	100.00%	1.00E−42	WP_060660918.1
*hp5*	Hypothetical protein	93.85%	9.00E−37	EAJ1438355.1
*hp6*	Hypothetical protein	100.00%	6.00E−135	WP_060660923.1
*hp7*	Hypothetical protein	100.00%	4.00E−143	WP_060660924.1
*hp8*	Hypothetical protein	100.00%	3.00E−144	WP_060662478.2
*hp9*	Hypothetical protein	100.00%	0.00E+00	WP_060662473.
*hp10*	Hypothetical protein	100.00%	1.00E−162	WP_060662474.1
*hp11*	Hypothetical protein			
*hp12*	Hypothetical protein	100.00%	9.00E−88	WP_005020456.1
*hp13*	Hypothetical protein	100.00%	8.00E−161	WP_054197458.1
*hp14*	Hypothetical protein	100.00%	0.00E+00	WP_104719495.1
*hp15*	Hypothetical protein	99.66%	0.00E+00	WP_060662472.1

## Discussion

In this study, based on the comparative genomic analysis, the comprehensive characteristics of these *H. pullorum* genomes were obtained. The results of core-pan genomes analysis showed highly intraspecies genomic diversity in *H. pullorum*. Human strain MIT98-5489 did not contain any specific genes. However, another human strain NCTC13156 from Canada had 99 specific genes, which also indicated that *H. pullorum* had a high genetic diversity. For three different groups, we found these three groups share 1821 genes, and the human source group contained less number of specific genes. Since the number of strains from each group were different, we cannot exclude the bias caused by the number of strains. The *cas1_2* gene (CRISPR-associated endonuclease Cas1) was the unique gene in two strains from human origin. In addition, there were 6 CRISPR-related genes in the pan-genome and found in all strains of human origin. The study of Marraffini et al. [15] showed that CRISPR loci counteracted multiple routes of HGT and could limit the spread of antibiotic resistance in pathogenic bacteria. Therefore, this might have an impact on the horizontal transfer of resistance genes.

2013BJHL was the first reported strain isolated from chicken liver in China. 76 specific genes were detected in this strain and four of them were related to antibiotic resistance. More drug resistance genes were found in the strains from Asia. For antimicrobial susceptibility testing, 2013BJHL showed the characteristics of multi-drug resistance. And antibiotic resistance genetic determinants on the 2013BJHL genome correlate well with observed antimicrobial susceptibility patterns. Resistance tests on 11 strains of *H. pullorum* in India have shown that all strains were multi-drug resistance [18]. Interestingly, in this study, the strains without individual missense mutations in the *gyrA* gene were isolated from European. We speculated that this might be related to the abuse of fluoroquinolone antibiotics in Asian settings. Moreover, all the antibiotic resistance genes in broiler chickens were AAC(6′)-Ie-APH(2′′)-Ia, and all the antibiotic resistance genes in free range chickens were APH(3′)-IIIa, and the number of antibiotic resistance genes in broiler chickens were more than that in free range chickens. This might be because in intensive farming, antibiotics were routinely fed to livestock as growth promoters and to prevent potential bacterial infections, which have contributed to increase in drug resistance worldwide, enabling re-emergence of zoonotic infections. At the same time, strains from different sources might have different patterns of drug resistance. These results also reflected the resistance situation of *H. pullorum* in Asia was more serious. All four strains were resistant to ciprofloxacin and two strains were resistant to erythromycin and tetracycline respectively in Borges’ study [4] and the perfect correlation between antimicrobial phenotypes and genotypes was found, which was also confirmed in our study. But our results were similar to those in India, showed characteristics of multi-drug resistance.

Core genome analysis played a key role in determining population structure which in turn shed light on the evolutionary trajectories of the strains [13]. The phylogenetic tree (Fig. 3) indicated the Asian strains were concentrated in Group1 and the Europe strains were concentrated in Group2. In addition, no drug resistance gene or gene individual missense mutation in the *gyrA* gene were detected in Group2. The genetic population structure of *H. pullorum* might be influenced by geographical distribution but due to the limitation of the analyzed strains the ecological niche characteristics could not be well inferred.

There was previous study reported that *H. pullorum* was capable of host cell adhesion, and the adhesion rate was comparable to *C. jejuni* [20]. Some homologous genes in *C. jejuni* were also found in *H. pullorum*. T6SS played an important role in the virulence of pathogens such as *Vibrio cholera* O1, *Salmonella* and *C. jejuni* [2, 10, 16]. In this study two subtypes of T6SS were identified. The existence of T6SS-related genes in all strains were consistent. The *tssA* gene was truncated in strain MIT98-5489, which might be due to the quality of the sequences, as there were lots of gaps in the regions of this strain. Although the interspecies region of these two subtypes of T6SS were not totally identical, the constituent genes showed highly similarities with the T6SS in *C. jejuni* [4]. The pathogenetic characteristics of these two subtypes of the T6SSs need further study.

## Conclusion

In a summary, this study we obtained the genetic characteristics of *H. pullorum* from different sources in the world. *H. pullorum* showed highly genetic diversity and two sub-types of T6SSs were first identified in *H. pullorum*.

## Materials and methods

### Sampling, isolation and identification of strain

2013BJHL was isolated from broiler retail chicken liver from Beijing, China in 2013. The isolate was grown on Campylobacter agar (OXIOD, UK) with 5% sheep blood in a microaerobic atmosphere (85% N_2_, 5% O_2_ and 10% CO_2_). After culturing, the DNA for genome sequence was extracted using the QIAamp DNA Mini Kit (Qiagen, German) according to the manufacturer’s instructions for sequencing. Then the NanoDrop spectrophotometer was used to measure concentration and purity of DNAs. The quality requirements were concentration ≥ 50 ng/μL and total amount > 20 μg. The purity requirement was as follows: OD260/OD280 value should be between 1.6 and 1.8.

### Antimicrobial susceptibility testing

Antimicrobial susceptibility to seven antibiotics (ciprofloxacin, nalidixic acid, tetracycline, gentamicin, streptomycin, erythromycin and ampicillin) was determined using the gradient strip diffusion method (E-test™, bio Mérieux, Nürtingen, Germany) following the manufacturer’s instruction. The bacterial suspension for the E-test was adjusted to 2 McFarland in 0.85% normal saline. 75 μl was evenly spread on a Campylobacter agar supplemented with 5% defibrinated sheep blood and a single strip was put on each plate. After 72 h of incubation at 42 °C under a microaerobic conditions, the minimum inhibitory concentration (MIC) was determined. The type strain of ATCC33560 was used as control. Thereafter, both the results were interpreted according to the Clinical and Laboratory Standards Institute (CLSI) recommendations.

### Whole genome sequencing (WGS) for strain 2013BJHL

The DNA sequencing was performed by an Illumina HiSeq2500Xten platform (Illumina Inc., San Diego, CA, USA) at the Beijing Genomics Institute (BGI) with a depth of 500× coverage. To sequence the genomes, a 270 bp paired-end library was constructed and then 150 bp reads were generated. FastQC (http://www.bioinformatics.babraham.ac.uk/projects/fastqc/) and fastp (https://github.com/OpenGene/fastp) software tools were applied to evaluate and improve the quality of the raw sequence data, respectively. Low quality reads were removed if the quality scores of ≥ 3 consecutive bases were ≤ Q30. The clean reads were assembled by SOAP*denovo* (http://soap.genomics.org.cn/soapdenovo.html). The assembled sequences were predicted to genes and annotated the function using Prokka pipeline [19] and glimmer software (http://ccb.jhu.edu/software/glimmer/index.shtml). Phage Search Tool (PHAST) web server(http://phaster.ca/) was used to search for phage sequences. The genome of strain 2013BJHL was submitted to NCBI, and the accession numbers was assigned as JXTX01000000.

### *Helicobacter pullorum* genomes from NCBI

In addition to *H. pullorum* 2013BJHL, the other 22 genomes were downloaded from NCBI (https://www.ncbi.nlm.nih.gov/) and PATRIC (https://patricbrc.org/). All isolates used in this study were listed in Table 2.

**Table 2:** Genomes of 24 *H. pullorum* genomes

Strain ID	Contigs	CDS	Source	Country	Assembly level	T6SS	GenBank accessions
NCTC13154	1	1668	Chicken caecum	UK	Complete Genome	–	LR134509
UBA1817	31	1666	Cecal contents	UK	Scaffold	–	DCEZ00000000
229254/12	138	1778	Fresh chicken meat	Portugal	Scaffold	+	JNOA01000000
229313/12	60	1657	Fresh chicken meat	Portugal	Scaffold	–	JNOB01000000
229334/12	229	2191	Fresh chicken meat	Portugal	Scaffold	+	JNOC01000000
229336/12	91	1766	Fresh chicken meat	Portugal	Scaffold	+	JNUR01000000
MIT98-5489	44	1926	Human	Canada	Scaffold	+	ABQU00000000
NCTC13156	3	1750	Human stool	Canada	Scaffold	–	UGJF01000000
NCTC12824	314	1894	Chicken faeces	Switzerland	Scaffold	+	VZPA01000000
35818/8	43	1603	Poultry feces	Belgium	Scaffold	–	FZMX01000000
35818/9	50	1793	Poultry feces	Belgium	Scaffold	+	FZMV01000000
NAP10B8	105	1632	Chicken caecum(broiler)	India	Scaffold	–	MAOZ00000000
NAP11B31	139	1819	Chicken caecum(broiler)	India	Scaffold	+	MAJF00000000
NAP12B32	130	1814	Chicken caecum(broiler)	India	Scaffold	+	MAJG00000000
NAP13B35	123	1811	Chicken caecum(broiler)	India	Scaffold	+	MANJ00000000
NAP14B36	101	1821	Chicken caecum(broiler)	India	Scaffold	+	MANK00000000
NAP1W4	162	1886	Chicken caecum	India	Scaffold	+	LXWI00000000
NAP2W5	140	1742	Chicken caecum	India	Scaffold	–	MAPE00000000
NAP3W17	74	1662	Chicken caecum	India	Scaffold	–	MAPD00000000
NAP5W19	127	1668	Chicken caecum	India	Scaffold	–	MAPC00000000
NAP6W24	108	1614	Chicken caecum	India	Scaffold	–	MAPB00000000
NAP8W25	134	1751	Chicken caecum	India	Scaffold	–	MAPA00000000
2013BJHL*	34	1855	Fresh chicken liver	China	Scaffold	+	JXTX01000000

“+”: with T6SS gene clusters, “−”:without T6SS gene clusters

### Core-pan genome analysis

Core-pan genome analysis was deduced using the Roary pipeline [17] with the.gff files from Prokka results. The parameters were chosen “-e (create a multiFASTA alignment of core genes using PRANK), -n (fast core gene alignment with MAFFT) and –v (verbose output to STDOUT)”. The genes contained in all strains were called core genes, and the genes contained in only one strain were called specific genes. The 3D map of the specific genes (Fig. 1) was drawn by using the scatterplot3d package. Then, the 23 strains were classified into three groups (human:2 strains, chicken feces:17 strains and chicken_meat:4 strains) according to the isolation source, and core-pan analysis was performed. Genes contained in all three groups were called core genes, and those that existed only in one group were considered specific genes. Venn diagram (Fig. 2) was drawn by using VennDiagram package to show the overlapping area of different element sets.

### Phylogenetic analysis

Core SNPs were called using Snippy4.3.6 software (https://github.com/tseemann/snippy) from the same reference (the complete genome NCTC13154). Gubbins software [7] was used as the recombination-removal tool to gain the pure SNPs without recombination. Phylogeny reconstruction was performed with the Maximum likelihood (ML) method using MEGA 7 software [11] with 1000 bootstraps.

### Virulence factors, antibiotic resistance genes and secretion system analysis

The virulence genes of all the genomes were detected on VFanalyzer (http://www.mgc.ac.cn/cgi-bin/VFs/v5/main.cgi?func=VFanalyzer). Resistance genes were predicted using the ResFinder (https://cge.cbs.dtu.dk/services/ResFinder/) and Comprehensive Antibiotic Resistance Database (CARD) (https://card.mcmaster.ca/?q=CARD/ontology/35506), with an E-value of at least 1e−10 as the cutoff. The identity cut-off and query coverage values were kept > 80% and > 60%, respectively. Blastn was used to detect individual missense mutation in *gyrA* gene, which was responsible for conferring ciprofloxacin resistance. The protein sequences were functionally annotated and classified using databases such as KEGG, COG, SwissProt and PHI et al. The nucleotide sequences of all the annotated T6SS-related genes were extracted from genomes, and related gene clusters were visualized by genoPlotR package. Heatmap of virulence genes was generated using the pheatmap package.

## **Supplementary information**

**Additional file 1.** Distribution of core-pan genes in *H. pullorum.*

**Additional file 2.** Distribution of gene presence and absence in three groups.

**Additional file 3.** Distribution of virulence genes in *H. pullorum.*

## Data Availability

The draft genome sequence of *H. pullorum* 2013BJHL has been deposited into GenBank database with accession number JXTX01000000, and the other 22 genomes were downloaded from NCBI (https://www.ncbi.nlm.nih.gov/) and PATRIC (https://patricbrc.org/).

## Abbreviations

*H. pullorum*: *Helicobacter pullorum*
T6SS: Type VI secretion system
WGS: Whole genome sequencing
CDS: Coding DNA sequences
*C. jejuni*: *Campylobacter jejuni*
ML: Maximum likelihood
BGI: Beijing Genomics Institute
SNP: Single nucleotide polymorphism
